# Dementia and Major Neurocognitive Disorders: Some Lessons Learned One Century after the first Alois Alzheimer’s Clinical Notes

**DOI:** 10.3390/geriatrics6010005

**Published:** 2021-01-11

**Authors:** Donatella Rita Petretto, Gian Pietro Carrogu, Luca Gaviano, Lorenzo Pili, Roberto Pili

**Affiliations:** 1 Department of Education, Psychology and Philosophy, University of Cagliari, Via Is Mirrionis 1, 09127 Cagliari, Italy; gpcarrogu@gmail.com (G.P.C.); luca.gvn@gmail.com (L.G.); LorenzoPili2011@gmail.com (L.P.); 2 Global Community on Longevity, Comunità Mondiale della Longevità, Selargius 09047, Italy; IERFOP Onlus, Cagliari, 09134 Presidenza@ierfop.org

Over 100 years ago, Alois Alzheimer presented the clinical signs and symptoms of what has been later called “Alzheimer Dementia” in a young woman whose name was Augustine Deter [1,2,3]. Alois Alzheimer described Augustine’s clinical history until her early death in 1906, and he focused his attention on a variety of impairments in functional domains in different phases of the disorder (first memory impairments, then delusions of husband’s adultery and sleeping disorders, then impairments in other cognitive domains and on consciousness). Alois Alzheimer studied her brain and described some peculiar clinical and pathological findings [1,2,3].

The word “dementia” was used for the first time by Pinel in 1797 [1,2], but there have been clinical notes on this disorder since ancient times. Some years later, the Alois Alzheimer’s clinical description of Augustine and after the endorsement of Kraepelin, who used for the first time the terminology “Alzheimer disease” in his 8th edition of Handbook of Psychiatry, dementia received a great attention from clinicians and from researchers. “Alzheimer Dementia” is now the most frequent and also the most known dementia all over the world [1,2,3].

Recently, international diagnostic criteria proposed to modify the name “dementia” with the string “major neurocognitive disorder” (MNCD) [4] aiming to reduce the negative stigma associated with the previous name and with his original meaning from the Latin word “demens” (“without mind”) [1]. Since the study of Alzheimer, MNCD gained a great attention in clinical and research field, and nowadays it is considered a major public health priority, due to its prevalence and incidence all over the world: over 6% in people older than 65 years, with an increasing of prevalence each five years of age and with an even higher prevalence in developing countries [5].

In this editorial, we aim to highlight some lessons learned in this field and to discuss some open questions since the first description of Alzheimer.

There are two main topics of interest: diagnosis/differential diagnosis, treatment and cure/care of people with MNCD. About diagnosis, in the last twenty years, there has been an increase of neuropathological findings and of neuroimaging findings at the base of the diagnosis of various forms of MNCDs, also with some overlapping between apparently different clinical conditions [6,7,8,9,10,11,12,13,14,15]. There has also been an increase of neuropsychological findings in different phases of the clinical history of various forms of MNCD [7,9,16,17,18,19,20,21]. As main consequences of these aspects, nowadays we know at least over 10 types of MNCDs and we know that there are at least three different kinds of clinical onsets: cognitive signs and symptoms, motor signs and symptoms and psychopathological/affective signs and symptoms [22,23]. There is now a general agreement about the role of recent and remote clinical history, the role of the description of onset signs and symptoms and the role of neuropsychological and psychopathological assessment [18,19,20,21,24]. Again, there is a general agreement on the need to correlate clinical manifestations with neuropathological and neuroimaging findings, even in the early phases of the disorder [7,16]. We also know more about the relationship between MNCD and mild neurocognitive disorders (previously named “mild cognitive impairment”), also with reference to the kind of onset signs and symptoms and with reference to clinical trajectories between them [22,23,25,26]. In recent years, differential diagnosis in MNCD has improved thanks to the integration of all currently available knowledge, but further research is still needed [22,23].

About the treatment, there is a general agreement on the need to integrate pharmacological and not pharmacological treatments and there are increasing data on the effectiveness of some non-pharmacological approaches, like those based on specific cognitive training [27,28,29]. As there are increasing findings on the overlapping between vascular MNCD and other kinds of MNCD, the roles of eating, health habits, diet and physical activities received great attention in longitudinal and retrospective studies in the field of primary and secondary prevention of MNCD [30,31]. Again, further research is needed also in the field of treatment.

There are still some open questions. Taking into account a worldwide approach, only 50% of people with MNCD received a correct diagnosis and adequate care and support; in developing countries, only 10% of people with MNCD are correctly supported [5]. International data showed an increasing prevalence of MNCD, mainly due to the relationship between constitutional/genetics factors [32,33,34], unhealthy habits [30,31] and negative environmental factors [35,36,37,38,39,40,41]. Moreover, the progressive ageing of the population has a central role in the increasing of prevalence, as the prevalence of MNCD is higher in higher ages [42]. Further research is needed on etiologies of MNCD and on the effectiveness of treatments and primary and secondary prevention interventions [5].

Moreover, taking into account individual experiences of people with MNCD, it is necessary to understand their unmet needs during the different stages of diseases, and it is mandatory to discuss how to increase and to support their quality of life and how to sustain their autonomy and independence [21,43,44,45,46,47,48,49,50,51]. There is still an open debate on the need to support people with MNCD in living in their homes and in their environments and, when they choose to live in medium and long-term facilities, it is important to support them also in this experience [51,52,53,54]. Negative experiences and risks related to institutionalization are debated, also taking into account the current experiences during the COVID-19 pandemic [55,56,57,58,59]. Moreover, the role of self-advocacy’s and families’ association is crucial. It is also necessary to better understand social costs on individuals and on other family members [60] and how to support caregivers and family members to adapt to caregiving and to prevent negative effects of family burden [5,54,61]. There also some ethical aspects related to the overall effect of MNCD on individual’s life and other ethical aspects related to advance care planning and end-of-life care [51,62,63,64].

In summary, in this editorial, we aim to discuss some lessons learned in the last century on MNCD, but we also propose some open questions that require higher attention in the next research in the field. Alzheimer showed great clinical comprehension, clinical intuition and foresight into detailing Augustine’s clinical history during her disorder: his clinical notes on the effects on different cognitive and behavioral domains of her life are consistent with the clinical approach confirmed by recent research. He has been a pioneer in this field. In about a century after the Alzheimer’s notes, clinicians and researchers have gained great knowledge in the field of MNCD and we believe that great work will be done in the near future. A trans-disciplinary approach, based on the cooperation between neurologists, neuroradiologists and other physicians, neuropsychologists and other health professionals, is crucial to gain more detailed knowledge in the field of MNCD, and the cooperation between different fields of study is mandatory. As MNCD affects all the domains of people’s life and it also affects different aspects of the Self and the project in life, we believe that a person-centered approach is needed, with a central role of the people with MNCD.
